# Protocol for development of a risk assessment tool for planning and management of religious mass-gathering events of India—a health system-strengthening initiative

**DOI:** 10.1186/s40814-019-0464-z

**Published:** 2019-06-24

**Authors:** Upasana Sharma, B. R. Desikachari, Sankara Sarma

**Affiliations:** 1 Achutha Menon Centre for Health Science Studies (AMCHSS), Sree Chitra Tirunal Institute for Medical Sciences & Technology (SCTIMST), Trivandrum, 695011 Kerala India; 2 Senior Public Health Consultant, Formerly with Department of Public Health and Preventive Medicine, Government of Tamil Nadu, Chennai, India

**Keywords:** Religious mass gathering planning and preparedness, Risk assessment tool, India mass gatherings, Community perspectives of mass gatherings, Mass gathering healthcare

## Abstract

**Background:**

Religious mass gatherings (MGs) have always been an integral part of our society. At the outset, mass-gathering events provide challenging settings to plan a suitable emergency public health response. Published studies basically talk about retrospective reviews, case studies of the public health preparedness, or health care provided at individual events. Developing an understanding of the variables associated with MGs is the first step for public health managers. Risk assessment (RA) is a crucial part of pre-event planning as it helps foresee potential risks. Based on RA, one can develop preventive measures and ensure that the infrastructure to control the potential problems is in place. This study is an attempt to systemize RA process during MG events in a country that is culturally rich but with poor resources to handle such events. A RA tool will be developed for planning and management of religious MG events of India.

**Methods/design:**

Various strategies will be used to develop the risk assessment tool (RA tool). Extensive review of literature clubbed with key informant interviews will be done in order to identify the risk variables and decide the domains and items of the tool. Further, this tool will be developed as a mobile-based application. The feasibility of the mobile-based RA tool will be tested in real-time MG event in one part of the country. Concurrently in the same event, a community survey of residents and visitors will be done in order to assess public perceptions of public health and environmental risks associated with MG events.

**Discussion:**

The findings of this study will provide insights into the public health and environmental concerns that need to be considered if preventive strategies and intervention programs are to be designed for MG events. A “RA Tool,” which can be used in the planning and management of MG events by the public health managers will strengthen the existing health systems preparedness plans for MGs.

## Background

According to the World Health Organization (WHO), a mass-gathering event (MG) is a gathering of persons that is usually defined as “the congregation of more than a specified number of people (this may be as few as one thousand persons; although most of the literature available, describes these as gatherings that exceed 25000 people) at a specific location for a specific purpose (a social function, a large public event, a sports competition) for a defined period of time” [1]. MGs can be either planned or spontaneous and recurrent or sporadic. Planned MGs may include sporting, social, cultural, religious, and political events. Examples include music festivals, the Olympic Games, the Hajj, and the Kumbh mela [1, 2]. Spontaneous MGs by their nature are more difficult to plan for and may include events such as funerals of religious and political figures [2]. MGs may also include the gatherings of displaced populations due to natural disasters, conflicts, and wars [1].

Such gatherings have several public health implications. There is increased risk of disease transmission because of huge influx of those attending the event. Overcrowding and overwhelmed medical services in such gatherings often aggravate the risk of infectious disease outbreaks [3]. Considerable challenges are posed by a mass gathering event (MG) in terms of communicable and non-communicable disease surveillance, emergency preparedness, environmental health, vaccination, crowd management, and various other issues [4]. In spite of the fact that MGs are an undeniably regular activity of our society that are attended by huge crowds, such gatherings are not very well understood. Even though such gatherings are accumulations of “well people,” vast number of people associated with MGs can put a serious strain on the entire health care system [3]. Along these lines, such MG events are more perilous and hazardous in terms of higher incidence of injury and illness compared to population in general [5]. Preplanning for MG events is crucial, and identification of potential health risks can be a vital element in pre-event planning for a MG [6]. Broadly, it includes health management and major incident planning [7]. Public health managers need to plan for, and respond to, a wide range of incidents and emergencies that could affect health or patient care. These could be anything from extreme weather conditions to an outbreak of an infectious disease or a major transport accident related to the MG event [1]. It is important to note that planning for MG events offer a chance to enhance health service delivery, strengthen public health systems, and escalate health promotion activities [8] but failure to plan sufficiently can also disturb the routine health services as the existing system is not attuned to deal with such gatherings of people [9]. The aim of public health at MGs is to prevent or limit the risk of injury or ill health and boost safety for participants, spectators, and residents of host community. Scope of planning for MG is largely dependent on the type of event, risk assessments (RAs), and available resources. Unfortunately, effective preparedness to mitigate and control health risks are inadequate when it comes to planning and management of MG events, especially in countries with poor resources [1]. It is important to note that public health priorities are determined based on the assumptions laid down during initial stages of public health planning of MGs [10]. For instance, in the development of any surveillance system, utilizing syndromic surveillance during MGs requires making suitable inquiries of the data before the event. Those inquiries are a result of RA, a process which has to be performed before and during the event in order to determine what public health-related outcomes will potentially occur and whether these outcomes can be addressed through syndromic surveillance [11]. RA serves as an initial step in the process of planning [12]. RA for a MG is a process that determines the intent and implementation of risk reduction measures, response planning, and capacity building for health functions. RA for MGs is undertaken to empower the public health authorities to identify and evaluate the generic characteristics of a MG which introduce or escalate specific threats. It incorporates assessment of the potential public health effects of the MG [1]. Systematic assessment of risks also helps to identify the potential health security risks that require cooperation of other departments and government agencies [8]. Existing research in the field of MG health is dedicated to the development of rapid diagnostic methods, monitoring and response, and treatment and vaccines. Public health surveillance systems are less likely to include information on non-infectious health threats such as air quality, which can be extremely important for event organizers. This area poses particular challenges for traditional public health surveillance which is generally designed to detect when things happen. This is where RA comes into picture [13]. For example, the leading causes of morbidity and mortality during the Hajj are heat-related illnesses and trauma-related injuries. Identification of such risks had allowed event planners to instigate preventive measures and rapid response strategies. For instance, provision of shaded areas reduced the incidence of heat-related illnesses and effective crowd control reduced the risk of a stampede. Drug and alcohol use were identified as health risks for other types of MGs [14]; therefore, restriction of their use mitigated the associated illnesses. In the context of limited resources, it has been suggested that one can choose to alleviate more likely events even if their potential impacts are smaller. Events that are catastrophic, but extremely unlikely, can then be given less priority [15]. During religious gatherings in India, some special events and unforeseen events occur at the places of religious MGs besides fixed places of worshipping. Special events like idol procession, chariot pulling, fire walking, and animal sacrificing happen pulling larger crowds within the MGs and causing more damage to human beings and property. History is in replete with incidences when MGs at fairs and festivals of India have turned into the hotspots of various types of risks [16]. Fire incidents have been reported to have occurred as a result of plastic sheets used as construction material for building cottages as temporary shelters for the pilgrims and also due to the use of liquid petroleum gas cylinders in the Kumbh mela premises and presence of unplanned and faulty electrical lines near lodging areas [17]. Previous studies [18, 19] have reported impaired water quality of Ganges River and consequential health risks by virtue of mass ritualistic bathing and insisted time and again that water of river Ganges was not fit for drinking or bathing purposes. A 10-year analysis of public health safety in 27 traditional MG events of India indicated around 936 dead and 540 injured casualties [20].

On examining the literature, we found that in the field of MG health, Paul Arbon has attempted to explain the process of RA using The Arbon Model. The Arbon technique is based on regression model that includes factors like attendance, seated vs mobile spectators, bounded vs unbound event, indoor vs outdoor event, sporting vs nonsporting event, humidity, and strictly daylight or night vs all day event and has been primarily used to define impacts on pre-hospital services [21].

Maurer’s formula has been used to define the quality and number of resources to be deployed at the time of event [22]. These conceptual models are based on the idea that MG health can be understood as an inter-relationship between three domains: (1) the biomedical, (2) the environmental, and (3) the psychosocial. Key features influence the rate of injury and illness and characterize each domain. These key features are more or less well understood and combine to produce an effect (the patient presentation rate) and a response (the health plan) [23].

According to the WHO [1], the process of RA can be accomplished in two ways, namely, strategic RA and case-based RA. The former method is undertaken before the event to thoroughly examine the potential risks. In addition to this, case-based RA may be required if a significant health concern is detected during the course of the event. It is worth mentioning that WHO has pointed out that event assessment and host country context assessment are extremely important for RA process [1]. A tool based on the modified version of British “Purple Guide” (a British guide for health, safety and welfare at music and other events) adjusted to Swedish context was developed with the aim to improve the quality of event’s planning. The reliability and validity of this tool was assessed on simulated case studies rather than real-life events [24]. A stratification scoring model was developed by Hartmann et al. [25] to predict resource use at the MGs. This model categorizes events into only three discreet severity types (minor, intermediate, major). Variables included are limited to attendance, heat index (if outdoor), presence of ethanol, crowd age, and crowd intention. For this, a retrospective observational study was conducted using records of 55 events of varying type. However, details of the various encounters during each event were not always available. Major shortcoming of this analysis was the inability to identify the individual impact of each of the predictive factors. To find a safe and easy method for the estimation of healthcare resources at sporting events, a template based on validated Swedish version of British event safety guide for music events was proposed by a team of researchers [26]. It was tested in six fictitious events. The authors concluded in their study that template could be used to estimate the healthcare resources in Sweden. However, they recommended that use of such a template requires some experience from previous RAs.

Here, it is pertinent to note that the foundation principles of the published studies with regard to RA have been the following: (1) based on availability of previous data, (2) tested on simulation case studies, (3) studies conducted in developed nations, (4) emphasis on medical care and medical resource estimation based on RA, and (5) nature of events in which the tools/models were tested focused on sports and music, leaving a huge scope of exploring the religious events.

Keeping in mind the end goal of planning and delivery activities at MGs, it is imperative to understand the MGs settings and RAs. Hence, in order to put the right measures in place to address the foreseeable and unforeseeable risks, the proposed RA tool will be fundamental in identifying potential public health risks and prioritize planning and response activities specific to the MG event. This activity will also be a step in the direction to establish a framework for a long-lasting national public health legacy. Against this backdrop, this study has been designed to achieve the following objectives:

1. To develop a risk assessment tool (RA tool) to assess the public health and environmental risks associated with religious mass-gathering (MG) events of Tamil Nadu
2. To develop a mobile application (app) based on the developed RA tool
3. To test the feasibility of the real-time application of the developed mobile app-based tool in a selected MG event of Tamil Nadu
4. To assess public perceptions of public health and environmental risks associated with MG event of Tamil Nadu

## Methodology

**Fig. 1:**
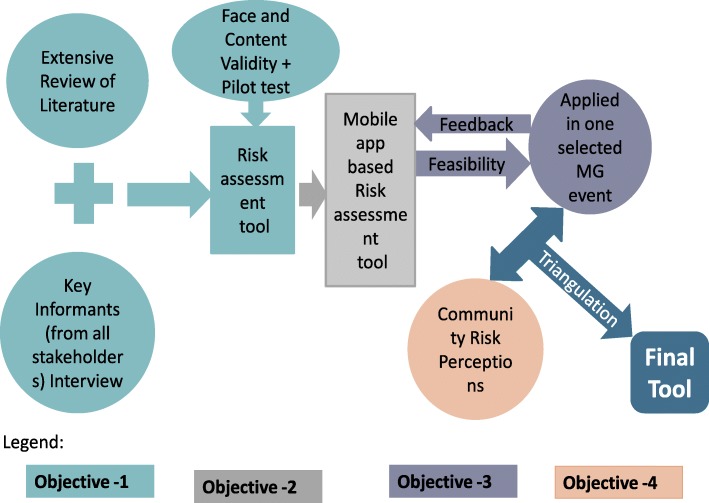
Study visual diagram. The file includes an overview of the study design

The research will be accomplished in various stages. The design of each stage has been described under relevant objectives. The following diagram represents the overall design of the research study (Fig. 1).

### Objective 1

The first aim of this study is to develop a RA tool to assess the public health and environmental risks associated with religious MG events in Tamil Nadu.

#### Study design

Qualitative study design will be elicited to develop the RA tool.

#### Study Methods

Key informant interviews and review of literature will be conducted to achieve the first objective of the study.

#### Key informant interviews

This include representatives from the Core Departments (Department of Public Health and Preventive Medicine, Department of Medical and Rural Health Services, Department of Police, Department of Fire and Rescue services, Department of Revenue and Disaster Management), officials from the Department of Veterinary Services, Community leaders, and NGOs, with experience in planning and management of MG event (at least one MG event). Academics and researchers with experience in MG and/or disaster management and/or scale development will be the subjects to be interviewed.

#### Review of literature

An extensive review of literature about the risks associated with the MGs, theoretical basis for RA and available RA tools for MG events, globally and nationally available RA tools, frameworks and guidance documents for MG will be done. The purpose of conducting the review of literature will be to identify the risks associated with the MGs reported in the previous studies. Identified health and environmental risks will be indicated in the RA tool. Identified risks will be categorized into relevant domains and items of the tool. A data extraction tool will be prepared by the researcher and used to extract information like study design, study area, place of study and other relevant details from the literature. Search strategy and database name will also be documented.

#### Study setting

Tamil Nadu, a state in the southern part of India, also known as the land of temples, will be the study site. It celebrates fairs and festivals round the year in all the districts with varying number of participants. Pilgrimage centers in Tamil Nadu are divided into “Places of Perennial Pilgrimage” and “Periodic Fairs and Festivals” [27]. Places of Perennial Pilgrimage are the ones which possess some special religious sanctity of their own apart from the occurrence of a holy day and which attracts pious persons in considerable numbers throughout the year, while places of fairs and festivals are those at which pilgrims congregate in numbers only on one or more special occasions during the year when the attraction may be either religious or secular or both. At places of periodic fairs and festivals, it is only at the time of the actual fairs and festivals that the place springs into importance and calls for special public health measures. Tamil Nadu is one of the states in India with best public health outcome indicators and one of the very few states with a dedicated public health care [28]. Public health has its own dedicated directorate in the State Health Department, in place since 1922 [29].The division has three key directorates which are hierarchically on an equivalent balance under the Health Secretary: the Directorates of Public Health, of Medical Services, and of Medical Education. At a district level, The Deputy Director of Health Services (DDHS) is responsible for the health of the district as a whole. DDHS broadly focuses on preventive and primary health care and is accountable to the Directorate of Public Health (DPH) for delivering both these types of service. DDHS manages all the workers at the district and block levels downwards who work on rural health, including all the staff of the Primary Health Centers and sub centers. However, in large municipalities, public health services are managed by municipal health officers (MHOs) nominated from the Directorate of Public Health as well. Usually, MHOs are supported by a staff of Sanitary Inspectors. Public health service arrangement in Tamil Nadu is greatly enabled by the fact that it has a Public Health Act. It gives the legislative foundation to Directorate of Public Health for policy planning and execution of public health activities. In Tamil Nadu, preparedness and management of MG events is guided by the Public Health Code (Part III Fairs and Festivals and Epidemics) and Tamil Nadu Public Health Act (1939). This document [27] lists around 123 fairs and festivals as “notified events” in the gazette for undertaking public health measures in the festival area.

#### Data collection

Key Informants involved in planning and management of MG events in the state of Tamil Nadu will be identified. We will conduct one to one semi structured interview among the identified key informants regarding their experiences, opinions, suggestions, risks, needs, and challenges associated with MG events. Once redundancy in responses is apparent, sampling recruitment will be ceased.

A semi-structured interview schedule will be used to guide the interview. The interview will be audio recorded. Transcript of each interview will be made on the same day. If and when needed, help of trained field investigators proficient in Tamil language to aid in language-related issues will be taken. KIs will be conducted in order to draw relevant domains and items from their real-time experience. Various risks identified through literature review and from discussion with KIs will be categorized under broad domains and sub domains or items.

#### Face and content validity of the developed tool

The face and content validity of the developed RA tool will be assessed by a group of experts (2–6 experts). Due care will be taken in selection of experts which will include public health experts, epidemiologists, academics with research experience in tool development, and government health administration officials. Using a self-administered content validity questionnaire, the experts will be asked to assess the relevance and representation of each item generated on a four-point Likert scale as 1 = not relevant, 2 = somewhat relevant, 3 = quite relevant, and 4 = highly relevant in order to avoid having a neutral and ambivalent midpoint. In a situation, where other kinds of validity are not feasible, content validity is an established and accepted method for validation in scientific community. Content validation methods focus on content relevance and content representation [30]. Multiple statistical measures of content validity like CVI and Kappa will be reported. To strengthen the validation exercise, the risks assessed by the tool will also be compared with the surveillance data of the MG event and the same will be reported.

#### Data analysis

Textual (content) analysis of key informant interviews will be done. Domains and items will be extracted from the textual data. Data collected from experts through content validity questionnaire will be entered and analyzed with the statistical software Epiinfo 7.1.5.2 version. Descriptive statistics like agreement proportions between the experts will be calculated. S-CVI (Scale Content Validity Index), index for inter-rater agreement (agreement proportion), and Kappa agreement coefficient will be calculated. Content validity index (CVI) is the most widely used index in quantitative evaluation [31] because it is simple for calculation, is easy to understand, and provide information about each item, which can be used for modification or deletion of instrument items. There are two kinds of CVI namely content validity of individual items (I-CVI), and the second is content validity of the overall scale (S-CVI). Researchers recommend [32] that a scale with excellent content validity should be composed of I-CVIs of 0.78 or higher and S-CVI/UA and S-CVI/Ave of 0.8 and 0.9 or higher, respectively. Kappa statistic is a consensus index of inter-rater agreement that adjusts for chance agreement [33] and is an important supplement to CVI because Kappa provides information about degree of agreement beyond chance [34].

#### Pilot test

The developed RA tool will be pilot tested among public health experts in a small MG of Tamil Nadu and appropriate changes will be made in the tool accordingly.

#### Expected benefits

Literature review along with the views of key informants’ will guide in identification of various public health and environmental risks associated with the MG events and a RA tool in its initial form will be developed.

### Objective 2

The second aim of this study is to develop a mobile application based on the developed RA tool.

#### Methods

An interdisciplinary team comprising of researcher, public health expert, an expert in scale development, and a software professional will be formed. Appropriate number of team meetings/discussions will be done. A smart phone-based mobile application will be developed based on the developed RA tool. Operational difficulties faced during the development of the mobile app will be documented.

- Content: developed RA tool
- Language: English
- Compatible Operating System: Android/iOS
- Target Audience: public health managers involved in planning and management of MG Events

#### Expected benefits

A smartphone-based mobile app based on the RA tool will be developed.

The mobile app will be based on the RA assessment tool. The intended audiences for the app are the public health managers who are involved in the preparedness activities of the MGs. The app will be used prior to the public event, i.e., pre-event stage by the experts to assess the potential risks that could be posed by the prospective event. The RA mobile app is expected to be useful for the following:

- Identify factors causing risk
- Avoid pitfalls and uncover opportunities
- Communicate risk to others
- Plan better strategies

The ultimate goal of the mobile app based on the RA tool is to identify and rank the most important factors driving the risks, so that the experts can plan strategies and resources accordingly. Based on the ranking, appropriate follow-up to diminish health risks of the event would be recommended based on the guidelines issued by organizations like WHO and other organizations of global importance. Here, a decision regarding what recommendations to include would be a part of the process of developing the RA tool.

### Objective 3

The third aim of this study is to test the feasibility of the application of mobile app based RA tool in a selected religious MG event of Tamil Nadu.

#### Study design

A cross-sectional study design will be employed to achieve this objective.

#### Study population

Public health preparedness team members of the selected religious MG event of Tamil Nadu, i.e., health officers, medical officers, epidemiologists, sanitary officers, Deputy Director of health services, entomologists, and representatives from fire and rescue services department, department of police, and department of revenue and disaster management will be the participants.

#### Study setting

A religious MG of perennial nature where larger crowds than usual foregather in the form of seasonal festivals notified by the government of Tamil Nadu will be selected as per convenience.

#### Sample size and sampling procedure

Representatives from various departments involved in public health planning and management will be purposively selected for the study.

#### Data collection

Representatives from the public health preparedness team will be briefed about the developed mobile app-based RA tool by the researcher. Selected members of public health preparedness team will be requested to use the developed mobile app-based RA tool in the selected MG event to assess the risks associated with the MG. Feedback regarding the mobile app and its content will be collected using a self-administered questionnaire. Operational difficulties faced during the application of the developed tool will be noted.

#### Data collection tools

Data will be collected using two tools: (i) The mobile application will be evaluated using a modified version of Mobile Application Rating Scale (MARS) for android-based mobile application. It is a self-administering mHealth app quality rating tool that provides a multidimensional measure of the app quality indicators of engagement, functionality, esthetics, and information quality as well as app subjective quality. It has been pilot tested on both iPhone as well as android apps [35]. (ii) Self-administered feedback questionnaire will be used to collect feedback on parameters like relevance, comprehensiveness, simplicity, and feasibility on Likert scale from the participants.

#### Data analysis

Data will be entered and analyzed with statistical software Epiinfo 7.1.5.2 version. Descriptive statistics like median and proportions will be used to express the ordinal data collected from the participants using the modified version of Mobile Application Rating Scale (MARS) and self-administered feedback questionnaire.

#### Expected benefits

Potential risks associated with selected MG event and state of planned control measures placed at the event site will be identified. Feasibility of application of the tool and operational difficulties faced during the use of mobile app-based RA tool in a real-time MG event will be assessed including the feedback of the participants regarding the mobile app. Feedback/suggestions/real-time experiences will help in further changes/modifications in the developed tool.

### Objective 4

The fourth aim of this study is to assess public perceptions of public health and environmental risks associated with MG event of Tamil Nadu.

#### Study design

A community-based cross-sectional study will be conducted to achieve the last objective of the research study.

#### Study population

Participants (outsiders attending MG event) and residents (food vendors, hotel staff, community members, etc.) of the host town, i.e., place of the MG event will be subjects of the community survey.

#### Study setting

Selected religious MG event of Tamil Nadu is the same event selected for objective no. 3.

#### Sample size

We assume that 50% of people attending a MG event perceive risks positively. With the relative precision of 10% and confidence interval of 95%, required sample size is calculated to be 384 (OpenEpi). With the assumption that 5% participants will be non-responders;,a total of 400 participants will be surveyed for the study.

#### Sampling procedure

Due to the absence of sampling frame, purposive quota sampling will be used. Multiple points in the event area will be selected, and at each point, participants will be selected randomly during different time points to achieve a representative sample.

#### Data collection instrument

A pre-designed and pre-tested semi structured questionnaire (translated into local language, i.e., Tamil) will be used to collect data.

#### Data collection

Data will be collected using the pre-designed and pre-tested semi-structured questionnaire to understand the risks perceived by the people attending the MG event. Help of appropriately trained field investigators will be taken to aid in language related issues.

#### Data analysis

Data will be entered and analyzed with statistical software Epiinfo 7.1.5.2 version. Mean and median will be computed to describe quantitative data. Proportions will be calculated for the categorical data. Inferential statistics like chi-square test will be used to compare the proportions between the groups and Student *t* test will be used to compare the means between the groups. Groups here indicate the comparison of perceptions between visitors and residents and other sociodemographic variables like gender, age group, educational status, employment status, and socioeconomic status.

#### Expected benefits

Risk perceptions, challenges, and problems faced by the people attending the selected MG event will be reported. Findings of this community survey will be triangulated with the mobile app findings, and based on that, tool will be finalized.

### Quality assurance

Field investigators recruited to aid the researcher in language related issues will be appropriately trained. The questionnaire used for community survey will be translated into local vernacular language and translated back to English by an independent translator, whose mother tongue is English and who has no knowledge of the questionnaire. Questions will be asked in the local vernacular language and responses will be recorded. The field investigators will be supervised periodically to ensure data completion and accuracy. The data will be double verified before entry and analysis.

## Discussion

This study aims to develop a RA tool consisting of characteristics peculiar to planned religious MGs of Indian context. This is an effort to systemize the process of RA by building upon the available guidelines and frameworks. A RA tool for planned MGs would be prepared that can be replicated across various religious gatherings of the state of Tamil Nadu and later across the country.

This will be achieved using a range of research methodologies namely key informant interviews and survey method. The novel RA mobile application developed during the course of the study will be field tested in a real-time religious MG event of the country.

Literature indicates [1–8] that we might be well equipped for response activities but the scientific concept of RA, i.e., to understand the existing risks, identify the risks, characterize the risks, and plan for risk reduction strategies accordingly, are at an infant stage in our country. The little that has been done in the field of MG has generally focused on description of preparedness activities of single event, crowd control, and prevention of stampedes with little attention to public health preparedness. It is pertinent to ask how current information and comprehension about MG events can be connected crosswise over various events. This study will take into account experiences, opinions, suggestions, risks, needs, and challenges associated with MG events by conducting one to one semi-structured interviews among the identified key informants. In addition, perspectives of the community, i.e., residents and visitors, will be explored and taken into account for the development of tool. This will help to understand community perspectives which will be useful to identify and understand the characteristics of MG event and also help to plan measures to address the risks identified.

As a part of the process of development of RA tool, community representatives like religious priest, community leaders, and members of civil society organizations will be included in key informants’ interviews. Thus, perspectives of the community will be explored and taken into account for the development of tool. The community (participants and visitors) risk perception survey will be conducted for triangulation purpose. Moreover, this survey may also provide insight into some “event specific” risks which could have not been considered by the researcher while developing the tool based on literature review and key informant interviews. The community risk perception survey will also provide information regarding difference in risk perception between the residents and visitors and difference in other sociodemographic indicators like gender, age, and socioeconomic status as well. Since it is a feasibility study, risk perception of the participants of MG events where the tool is applied is necessary to improve the tool further till it reaches the stage of complete validation.

This research will hence contribute to public health preparedness activities of MG events in a country like India with infrastructure limitations. The size and number of MG events in the future will continue to increase, and hence, this emerging area of MG health demands research for the safe conduct of MGs. The deliverables of this research, i.e., RA tool in the form of mobile application, can be used in the planning and management of MG events by the public health managers.

## Limitations

Due to resource constraints, the developed risk assessment (RA) tool will be field tested in a single religious MG event. Since religious MGs are diverse in nature, each MG is unique in itself. So, it is advisable to test the RA tool in multiple MG events to ensure its validity. This study will serve as an initial step towards systemizing the process of RA in the state of Tamil Nadu giving enough scope to fine tune it in future. The novel RA tool that will result from this research study will be limited to risk identification and its characterization. It is recognized that further research studies will be needed to add on components like risk-wise recommendations and resource estimation to the RA tool.

## Abbreviations

CDC: Centre for Disease Control
DDHS: Deputy Director of Health Services
I-CVI: Item-Content Validity Index
MARS: Mobile Application Rating Scale
MG: Mass gatherings
MHO: Municipal Health Officers
NDMA: National disaster management authority
NGO: Non-governmental organization
PHP: Public health preparedness
RA: Risk assessment
S-CVI/Ave-Scale: Scale-Content Validity Index/Average
S-CVI/UA-Scale: Scale-Content Validity Index/Universal Average
WHO: World Health Organization

## Authors’ information

US is supported by the Indian Council of Medical Research (ICMR) Senior Research Fellowship.
